# Improving Timely VTE Risk Assessment in Adult Psychiatric Inpatients: A Quality Improvement Project

**DOI:** 10.1192/bjo.2026.11268

**Published:** 2026-06-30

**Authors:** Oluwagbemiga Adesina

**Affiliations:** Lancashire and South Cumbria NHS Foundation Trust, Preston, United Kingdom

## Abstract

**Aims:**

To improve compliance with Trust and NICE guidance requiring completion of venous thromboembolism (VTE) risk assessments within 24 hours of admission for adult psychiatric inpatients.

**Methods:**

Baseline data were collected in May, 2025 from 20 inpatients to assess timeliness and documentation of VTE risk assessments following admission. Electronic records (RiO and NerveCentre) were reviewed to determine whether assessments were completed within 24 hours and whether reasons for delay were documented.

Following baseline measurement, a series of targeted quality improvement interventions were introduced. These included ward poster reminders, education during handovers and MDT meetings, VTE training incorporated into resident doctor inductions, routine MDT review of VTE status, addition of a VTE column to doctors’ handover sheets, ward-specific completion protocols, and reinforcement of documentation standards.

Post-intervention data were then collected retrospectively in November 2025 for 13 inpatients admitted after implementation of these measures. The same outcome measures were analysed to assess improvement.

**Results:**

Overall VTE assessment completion was 100% at both baseline and post-intervention. Timely completion within 24 hours improved from 50% (10/20) at baseline to 84.6% (11/13) post-intervention. Delayed assessments decreased from 50% to 15.4%. Documentation of reasons for delay improved from 20% to 100%. Mean time to assessment reduced substantially from 36 hours to 11.6 hours. Documented reasons for delay included patient refusal and the patient being asleep at the time of assessment.

**Conclusion:**

Targeted, low-cost quality improvement interventions led to marked improvement in the timeliness and documentation of VTE risk assessments in an adult psychiatric inpatient ward. Although the NICE target of >95% completion within 24 hours has not yet been achieved, substantial progress was demonstrated. Sustained education, system prompts, and MDT oversight are expected to support further improvement and enhance patient safety.

